# Novel next generation sequencing panel method for the multiple detection and identification of foodborne pathogens in agricultural wastewater

**DOI:** 10.3389/fmicb.2023.1179934

**Published:** 2023-07-13

**Authors:** Dong-Geun Park, Joon-Gi Kwon, Eun-Su Ha, Byungcheol Kang, Iseul Choi, Jeong-Eun Kwak, Jinho Choi, Woojung Lee, Seung Hwan Kim, Soon Han Kim, Jeongwoong Park, Ju-Hoon Lee

**Affiliations:** ^1^Department of Food and Animal Biotechnology, Seoul National University, Seoul, Republic of Korea; ^2^Department of Agricultural Biotechnology, Seoul National University, Seoul, Republic of Korea; ^3^Research Institute of Agriculture and Life Sciences, Seoul National University, Seoul, Republic of Korea; ^4^Center for Food and Bioconvergence, Seoul National University, Seoul, Republic of Korea; ^5^Research and Development Center, Sanigen Co., Ltd, Anyang, Republic of Korea; ^6^Division of Food Microbiology, National Institute of Food and Drug Safety Evaluation, Ministry of Food and Drug Safety, Cheongju, Republic of Korea

**Keywords:** next-generation sequencing, NGS panel, foodborne pathogen, multiple detection, real-time PCR

## Abstract

Detecting and identifying the origins of foodborne pathogen outbreaks is a challenging. The Next-Generation Sequencing (NGS) panel method offers a potential solution by enabling efficient screening and identification of various bacteria in one reaction. In this study, new NGS panel primer sets that target 18 specific virulence factor genes from six target pathogens (*Bacillus cereus*, *Yersinia enterocolitica*, *Staphylococcus aureus*, *Vibrio cholerae*, *Vibrio parahaemolyticus*, and *Vibrio vulnificus*) were developed and optimized. The primer sets were validated for specificity and selectivity through singleplex PCR, confirming the expected amplicon size. Crosscheck and multiplex PCR showed no interference in the primer set or pathogenic DNA mixture. The NGS panel analysis of spiked water samples detected all 18 target genes in a single reaction, with pathogen concentrations ranging from 10^8^ to 10^5^ colony-forming units (CFUs) per target pathogen. Notably, the total sequence read counts from the virulence factor genes showed a positive association with the CFUs per target pathogen. However, the method exhibited relatively low sensitivity and occasional false positive results at low pathogen concentrations of 10^5^ CFUs. To validate the detection and identification results, two sets of quantitative real-time PCR (qPCR) analyses were independently performed on the same spiked water samples, yielding almost the same efficiency and specificity compared to the NGS panel analysis. Comparative statistical analysis and Spearman correlation analysis further supported the similarity of the results by showing a negative association between the NGS panel sequence read counts and qPCR cycle threshold (Ct) values. To enhance NGS panel analysis for better detection, optimization of primer sets and real-time NGS sequencing technology are essential. Nonetheless, this study provides valuable insights into applying NGS panel analysis for multiple foodborne pathogen detection, emphasizing its potential in ensuring food safety.

## Introduction

Foodborne pathogens, including *Escherichia coli* O157:H7, *Salmonella, Bacillus cereus, Yersinia enterocolitica, Staphylococcus aureus,* and *Vibrio*, are widespread and frequently cause foodborne diseases. In the USA from 2009 to 2020, 9,720 foodborne pathogen associated disease outbreaks occurred, causing 168,656 illness, 10,983 hospitalizations, and 268 deaths (Lee and Yoon, 2021). In South Korea from 2010 to 2018, there were 2,815 outbreaks of foodborne and waterborne diseases, which posed health risks to the population (Lee et al., 2021). To prevent or reduce such serious foodborne disease outbreaks, it is necessary to rapidly detect foodborne pathogens; thus, the development of efficient foodborne pathogen detection methods is essential (Lee et al., 2001).

Foodborne pathogen detection methods can be divided into four types: (1) culture-based detection, (2) immunological detection, (3) biosensor-based detection, and (4) DNA-based detection. Culture-based detection is the traditional foodborne pathogen identification method; thus, it has a long history and is considered the gold standard (Bhunia, 2014). Using this method, viable colony forming units (CFUs) of foodborne pathogens are detected in genus-specific selective media cultures, and live CFUs and cell numbers can be confirmed in contaminated samples cost-effective and well-established manner (Bolton, 1998). However, at least 2–3 days are required to obtain the results of culture-based foodborne pathogen detection tests, and these are followed by biochemical tests, molecular tests, and/or mass spectrometry (Zhao et al., 2014). Therefore, alternative rapid foodborne pathogen detection methods have been developed. Immunological detection involves the use of an antibody–antigen reaction to detect foodborne pathogens; the methods used include enzyme-linked immunosorbent assays (Fusco et al., 2011), lateral flow immunoassays (Shi et al., 2015), and immunomagnetic separation assays (Shim et al., 2008). Monoclonal or polyclonal antibodies are used for different specificities to detect specific antigens, offering rapid, portable, and economic detection in the commercial ELISA-based detection kit via their massive production (Umesha and Manukumar, 2018). However, the influence of environmental stress on the antibody leads to low accuracy in immunological detection (Hahn et al., 2008). Biosensor-based detection was developed to overcome the disadvantages of immunological detection. Specifically, optical piezoelectric biosensors have been developed that provide a wide working range, rapid results, portability, and enhanced detection accuracy and limit of detection (Velusamy et al., 2010). Therefore, biosensors for bacterial quantification are generally rapid, specific, sensitive and very reliable. However, the development and commercialization cost of biosensors are relatively high, including the production of inexpensive sensors, storage and stabilization of biosensors, calibration methods, and achieving complete integration of the sensor system. Once developed and optimized, the cost of biosensors for bacterial quantification can be lowered (Tokarskyy and Marshall, 2008; Nnachi et al., 2022). DNA-based detection via specific gene-based polymerase chain reaction (PCR) is generally used in foodborne pathogen diagnostics in laboratories (Priyanka et al., 2016). Because of DNA amplification, conventional PCR in which specific gene-targeting primers are used exhibits high sensitivity up to the femtogram level (Palka-Santini et al., 2009). However, this method still requires a time-consuming electrophoresis step for the detection and confirmation of specific genes, and only one gene can be detected in each analysis (Joensen et al., 2014). To overcome the limitations of conventional PCR, real-time PCR or multiplex PCR methods were developed and optimized. Real-time PCR using specific gene-targeting primers and a probe does not require the electrophoresis step, and specific genes can be detected via the fluorescence signal from the probe (Yang et al., 2015). Determining fluorescence intensity also enables the quantification of DNA concentrations (Liu et al., 2019). Multiplex PCR can detect a few targeted genes at the same time because a mixture of primer sets is used (Chen et al., 2012). Combining these advantages, multiplex real-time PCR was developed. Many PCR-based detection kits developed in recent years use multiplex real-time PCR, which achieves rapid and multiple detection with high specificity and sensitivity (Park et al., 2020). Next-generation sequencing (NGS) has enabled the generation of large quantities of DNA sequences in an economical and time-efficient manner (Gupta and Verma, 2019). The most frequently used NGS sequencers are those from Illumina, which provide the prevailing high-throughput technology with the highest fidelity (Yohe and Thyagarajan, 2017). Although NGS produces massive amounts of DNA sequences in one run, the technology was highly expensive at the early stage (De Magalhães et al., 2010). However, NGS services have been popularized and subject to reduced costs given the continuous development of new technologies such as nanopore (Oxford NanoPore Technologies, United Kingdom) sequencing (Vega et al., 2016). Given the reduced costs, this NGS sequencing service is now available for use in molecular studies of foodborne pathogens to achieve rapid detection and identification and facilitate microbial genomics, metagenomics, and even shotgun metagenomics analyses (Chung et al., 2021). The term “NGS panel” refers to an NGS-based assay that allows for the simultaneous analysis of multiple genes, genetic variants, microbial genomes, or other genomic features. In particular, NGS panels are promising analysis methods with which hundreds to thousands of target gene sequences can be screened at once and many samples can be simultaneously analyzed to rapidly and efficiently detect and identify foodborne pathogens (Ferrario et al., 2017).

The NGS panel method was initially evaluated and used in clinical cancer diagnoses and genetically modified organism (GMO) determination. In a previous study, an NGS panel with 13 endometrial cancer gene target primers was developed and evaluated, and 20 randomly chosen cases of patients with endometrial cancer were successfully classified, highlighting the rapid and accurate diagnosis ability of NGS panels (López-Reig et al., 2019). In another study, a NGS panel with four GMO-related target gene sequences was developed and evaluated using real-time PCR as the control; the NGS panel and real-time PCR provided a 92% GMO detection rate, indicating the reliability of screening performed via this method (Arulandhu et al., 2018). Given the advantages of NGS panels, they have also been evaluated and tested for the multiple detection and determination of various foodborne pathogens. Prior to the use of NGS panels, the detection and identification of foodborne pathogens was conducted using 16S rRNA sequencing based on Sanger sequencing, 16S rRNA-based metagenome and random genome sequencing-based shotgun metagenomics approaches (Bridier, 2019); however, these detection methods produce an overabundance of sequence information (Zakotnik et al., 2022). To overcome this problem, NGS panels were developed and evaluated using specific primer sets, generally targeting the virulence factors and antibiotic resistance genes of foodborne pathogens. However, only one NGS panel study has involved the detection and identification of multiple foodborne pathogens; in this study, a species-specific multiplex PCR amplicon was sequenced using an Illumina MiSeq sequencer to a sensitivity of 10^1^ CFUs/g (Ferrario et al., 2017). This study demonstrates that, compared with metagenome and shotgun metagenomics sequencing, the NGS panel approach achieves rapid and accurate species-specific identification via the one-time compact NGS sequencing of virulence factors and antibiotic resistance genes. Only one primer set per pathogen was used in this study, and the specificity and sensitivity of the primer sets were not fully evaluated; however, the importance of NGS panel primer set quality and the requirement of multiple primer sets per pathogen should be considered. Indeed, the NGS panel method should be optimized with reliable multiple primer sets.

In the present study, we aimed to optimize the NGS panel method for the detection and identification of six major foodborne pathogens in South Korea: *Bacillus cereus*, *Yersinia enterocolitica*, *Staphylococcus aureus, Vibrio cholerae, Vibrio parahaemolyticus,* and *Vibrio vulnificus*. In addition, 2–5 species-specific primer sets per pathogen were designed and evaluated. With these new primer sets, the NGS panel method was tested and evaluated using the six selected foodborne pathogens. To verify the sensitivity and accuracy of the NGS panel, multiplex real-time PCR was performed as a control and compared with the NGS panel results. This study provides a novel optimized NGS panel method that achieves the rapid and accurate detection and identification of selected foodborne pathogens in contaminated samples with efficiency, sensitivity, and accuracy. Therefore, this technology could be useful for ensuring food safety through the prevention of foodborne disease outbreaks via the rapid and accurate detection and identification of foodborne pathogens.

## Materials and methods

### Bacterial strains, selective/culture media, and growth conditions

The bacterial strains and selective/culture media used in this study are listed in Table 1. All bacterial strains were aerobically incubated at 37°C for 18 h. All culture media were purchased from Oxoid (United Kingdom), and the agar medium was prepared with 1.8% BACTO Agar (BD, United States).

**Table 1:** Bacterial strains, culture medium, samples, and sampling locations.

Bacterium	Strain	Selective media^a^	Culture media^b^	Reference^c^	Sample	Sampling location
**Selected foodborne pathogens**						
*Bacillus cereus*	SG_003	BBC	LB	This study	Seaweed fulvescens	Garak Agricultural and Fisheries Wholesale Market, Seoul
*Yersinia enterocolitica*	SG_002	CIN	LB	This study	Pollack roe	Garak Agricultural and Fisheries Wholesale Market, Seoul
*Staphylococcus aureus*	ATCC 23235	-	LB	ATCC	-	-
	Newman	-	LB	ATCC	-	-
	CCARM 3089	-	LB	CCARM	-	-
	SG_001	MSA	LB	This study	Crab	Garak Agricultural and Fisheries Wholesale Market, Seoul
*Vibrio cholerae* (non-O1-type)	SG_017	TCBS	LB	This study	Octopus	Noryangjin Seafood Wholesale Market, Seoul
*Vibrio vulnificus*	SG_012	TCBS	LB	This study	Mussel	Noryangjin Seafood Wholesale Market, Seoul
*Vibrio parahaemolyticus*	SG_014	TCBS	LB	This study	Sea urchin	Noryangjin Seafood Wholesale Market, Seoul

a MSA, mannitol salt medium; CIN, cefsulodin–irgasan–novobiocin medium; BBC, Brillance *Bacillus cereus* medium; TCBS, thiosulfate–citrate–bile salts–sucrose medium.

b LB, Luria–Bertani medium.

c ATCC, American Type Culture Collection; CCARM, Culture Collection of Antimicrobial Resistance Microbes.

### Isolation of foodborne pathogens

For the isolation of foodborne pathogens, five seafood samples were collected from Garak Fisheries Wholesale Market (Seoul, Korea) and Noryangjin Seafood Wholesale Market (Table 1). After sample collection, 25 g of the collected samples were transferred to a 3 M sterilized bag (USA) and suspended with 225 mL of sterilized phosphate-buffered saline buffer. Suspended samples were homogenized using a BagMixer 400 (Interscience, France) with a speed of 4 m/s for 30 s. After homogenization, the samples were serially diluted to 10^−6^, plated on selective agar plates specific for each pathogen (Table 1), and incubated as described previously. From each selective agar plate, multiple colonies were picked and each colony was separately inoculated into fresh broth culture media. After broth culture incubation, the selected bacterium was identified using 16S rRNA gene sequencing technology (Table 1). The selected bacterium was identified using 16S rRNA gene sequencing technology, and the identified bacterium was stored at −80°C in 10% (w/v) sterilized skim milk solution.

### DNA extraction

Bacterial genomic DNA was extracted and purified using a Genelix™ Bacterial Extraction Kit (Sanigen, South Korea) according to the manufacturer’s instructions. In preparation for NGS panel analysis, total bacterial DNA was extracted from prepared agricultural water samples spiked with the six selected foodborne pathogens or agricultural water free of these pathogens using a QIAamp DNA Stool Mini Kit (Qiagen, United States) according to manufacturer’s standard protocol.

### 16S rRNA gene sequencing

All PCRs were performed using a C1000 Touch Thermal Cycler (Bio-Rad, United States). In addition, 16S rRNA gene sequencing was performed for bacterial identification under the following conditions. The PCR mixture (final volume: 25 μL) contained 1 μL of template DNA (40 ng/μl), 0.5 μL of forward primer (20 μM; 27F, 5′-AGAGTTTGATCCTGGCTCAG-3′) and 0.5 μL of reverse primer (20 μM; 1492R, 5′-GGTTACCTTGTTACGACTT-3′; Montagner et al., 2010), 12.5 μL of BioFACT™ 2X Taq PCR Master Mix (BioFact, South Korea), and 10.5 μL of molecular water. The PCR conditions were as follows: 1 cycle of 95°C for 3 min; 35 cycles of 95°C for 30 s, 60°C for 30 s, and 72°C for 30 s; and 1 cycle of 72°C for 5 min. Following PCR, 16S rRNA amplicons were purified using a NICSROprep™ PCR Clean-up S and V Kit (Bionics, South Korea) and sequenced using a 3730xl DNA Analyzer (Thermo Fisher, United States) at Bionics in South Korea according to manufacturer’s standard protocols.

### Genome sequencing and analysis

For sequencing library preparation with the bacterial genomic DNA, a TruSeq Nano DNA LT Kit (Illumina, United States) was used to add sequencing barcodes to NGS sequencing templates. The sequencing library was then sequenced using an Illumina MiSeq system according to the Illumina MiSeq 2 × 150 bp paired-end run protocol. The qualified sequence reads were assembled using the Unicycler program (Wick et al., 2017) and the assembled contigs of each foodborne pathogen were annotated using the NCBI Prokaryotic Genome Annotation Pipeline (Tatusova et al., 2016).

### NGS panel primer design and optimization

The publicly available complete genome sequences of target pathogens were collected from the GenBank database in the NCBI.^1^ Comparative pan-genome analysis with the complete genome sequences of other pathogens was performed using the panX program (Ding et al., 2018) to identify target pathogen-specific genes. Among the detected pathogen-specific genes, virulence factors and antibiotic resistance genes were primarily considered for selection. New primer sets for the NGS panel were then designed using the sequences of the selected genes and the Primer3 program (Untergasser et al., 2012) with the following parameters: size: 100–300 bp; GC content: 40%–60%; T_m_ value: 53°C–60°C; self-compatibility: ≥4. After primer set design, the stability of the primers, e.g., self-binding and dimer formation, and specificity of the primer set to the target pathogen genome sequence were confirmed using Primer3. For NGS panel sequencing analysis, 2–5 genes per target pathogen were selected for primer design. Therefore, a single pathogen had 2–5 specific primer sets, and each primer set was optimized as previously explained. The selected pathogen-specific genes and their targeting primer sets are listed in Supplementary Table S1.

### Singleplex PCR and crosscheck PCR

To validate the primer specificity to the target pathogen genome sequence, singleplex and crosscheck PCRs were performed. For singleplex PCR, the PCR mixture (final volume: 25 μL) contained 1 μL of template DNA (4 ng/μL), 0.5 μL of forward and reverse primers (20 μM), and 12.5 μL of KAPA HiFi HotStart ReadyMix (Roche, Germany), and the final volume was adjusted with molecular water. Crosscheck PCR was used to evaluate the selected primer set, and two approaches were taken: (1) a single primer set with the genomic DNA of 9 target strains and (2) multiple primer sets (2–5 primer sets per reaction) with the genomic DNA of a single target strain. The PCR mixture of the first crosscheck PCR test was prepared with the same composition as that used in singleplex PCR, except for the genomic DNA templates. The test genomic DNA template mixture for the first crosscheck PCR was prepared with the genomic DNA of a target pathogen and other nontarget pathogens, and the negative control genomic DNA mixture was prepared with the genomic DNA of only the nontarget pathogens. These template DNA mixtures contained 4 ng/μL of DNA per pathogen. The PCR mixture of the second crosscheck PCR test had the same composition as that used in the singleplex PCR, except for the multiple primer sets, which themselves contained 2–5 primer sets (20 μM of each) per reaction with a single target strain. The PCR conditions for both the singleplex and crosscheck PCRs were as follows: 1 cycle of 95°C for 3 min; 35 cycles of 95°C for 30 s, 60°C for 30 s, and 72°C for 30 s; and 1 cycle of 72°C for 5 min. To verify the PCR results, agarose gel electrophoresis was performed with 2.5% agarose gel containing ethidium bromide (0.2 μg/mL), and the size of each PCR amplicon was confirmed in the gel using 100 bp DNA ladder (Bioneer, South Korea) after the gel was run at 135 V for 20 min.

### Multiplex PCR

In addition to singleplex and crosscheck PCRs, multiplex PCR was performed to confirm the specificity of the primer sets in the multi-detection of target pathogens. The multiplex PCR mixture (final volume: 25 μL) contained 1 μL of template DNA (4 ng/μL per pathogen; 9 pathogens in total), 0.5 μL of forward and reverse primer sets (2–5 primer sets; 20 μM of each), and 12.5 μL of KAPA HiFi HotStart ReadyMix (Roche, Germany), and the final volume was adjusted with molecular water. The test and negative control genomic DNA mixtures were prepared with the same composition as that used in the first crosscheck PCR. The PCR results were verified following the same procedure used in singleplex and crosscheck PCRs.

### Collection of agricultural water, and the preparation of simulated agricultural water samples with selected pathogens

Six agricultural water samples were collected from a vegetable farm in Hadong-gun, Gyeongsangnam-do, South Korea. Four samples, namely B4GNG1-1 (chive), B4GNG4-2 (chive), B1GNG8-1 (cabbage), and B1GNG8-2 (cabbage), were collected from ground water, whereas two samples, B1GNS10-1 (cabbage) and B1GNS10-2 (chive), were collected from stream water. One liter of each water sample was collected and transferred into a 2-L sterilized water pack (Worldmedi, South Korea). For the preparation of the spiked water samples, nine pathogenic strains were selected as follows: *V. vulnificus* SG_012; *V. parahaemolyticus* SG_014; non-O1-type *V. cholerae* SG_017; *Y. enterocolitica* SG_002; *S. aureus* strains SG_001, ATCC 23235, Newman, and CCARM 3089; and *B. cereus* SG_003 (Table 1). Six selected foodborne pathogenic strains and three reference strains were separately inoculated into fresh Luria–Bertani (LB) media and incubated up to 1.0 optical density at a wavelength of 600 nm. Subsequently, the CFUs of each culture was adjusted to 1.0 × 10^8^ CFUs/mL using sterilized LB broth medium. To prepare a single *S. aureus* culture containing four different strains, 25% of each *S. aureus* strain culture was mixed. The negative control was prepared with an agricultural water sample without inoculation of target pathogens. Each CFU-adjusted culture of a selected pathogen was mixed, and the culture mixture containing six selected pathogenic species was centrifuged at 13,000 rpm for 10 min to harvest the bacterial cell mixture (1.0 × 10^8^ CFUs per pathogen). This mixed cell pellet (6.0 × 10^8^ CFUs) was resuspended using 250 mL of each collected agricultural water sample. The resuspended bacterial mixture was then serially 10-fold diluted to 6.0 × 10^5^ CFUs per sample (1.0 × 10^5^ CFUs per target pathogen in the sample). These serially diluted agricultural water samples (10^8^, 10^7^, 10^6^, and 10^5^ CFUs per target pathogen) were used for total bacterial DNA extractions before further NGS panel analysis. This experiment was performed in triplicate.

### NGS panel analysis

To prepare the NGS panel sequencing template DNA via PCR, two sets of template DNA were prepared: (1) total DNA for test samples from one of six agricultural water samples containing target pathogens and (2) total DNA for negative controls from one of six agricultural water samples without the addition of target pathogens. The PCR mixture (final volume: 25 μL) contained 1 μL of template DNA (the total DNA template for test samples or total DNA template for negative controls), 0.1 μL of forward and reverse primers per primer set (18 primer sets; 100 μM of each), and 12.5 μL of KAPA HiFi HotStart ReadyMix (Roche), and the final volume was adjusted with molecular water. The PCR conditions used were the same as those used in singleplex PCR. Following PCR, target PCR amplicons were gel-extracted and purified using a NICSROprep™ DNA Gel Extraction S & V Kit (Bionics) according to the manufacturer’s standard protocol. To prepare the sequencing library, a TruSeq Nano DNA LT Kit (Illumina, United States) was used to add sequencing barcodes to NGS sequencing templates. Subsequently, the sequencing library was sequenced using an Illumina MiniSeq system according to the Illumina MiniSeq 2 × 150 bp paired-end run protocol. After NGS sequencing, the following steps were taken: (1) a filtering step in which the raw reads were filtered using Trimmomatic (Bolger et al., 2014) to obtain a Phred quality score of >20; (2) a merging step in which the filtered reads were merged using Pandaseq (Masella et al., 2012) with its default parameters; and (3) a mapping step in which the merged reads were mapped to the six selected pathogen-specific gene sequences using BLASTN with a >95% nucleotide identity (Camacho et al., 2009). Finally, the number of mapped reads was counted. The detection criteria for false positive (<6 read counts) was determined using NGS panel analysis with negative controls, the agricultural water samples without inoculation of target pathogens. The false positive was determined using this detection criteria after counting the number of read counts from the NGS panel analysis result.

### Quantitative real-time PCR

To evaluate the NGS panel analysis, quantitative real-time PCR (qPCR) was performed, and the results were compared with the NGS panel analysis results. The qPCR was performed using a CFX96 deep-well plate reader (Bio-Rad). The two sets of NGS panel sequencing template DNA previously described were used as the template DNA for qPCR. A Genelix™ Multiplex Real-Time PCR Kit (#G102, Sanigen) was used to detect *V. vulnificus, V. parahaemolyticus*, and *V. cholera*, whereas a Genelix™ Multiplex Real-Time PCR Kit (#G104, Sanigen) was used to detect *B. cereus, Y. enterocolitica*, and *S. aureus*. The qPCR was performed according to the manufacturer’s standard protocols, and the Ct was determined automatically using CFX Manager Software version 3.1 (Bio-Rad). All tests were performed in triplicate.

### Statistical analysis

GraphPad version 7.0 (Prism, United States; http://www.graphpad.com) and R version 4.1.2 (R Core Team, 2013) were used to perform all correlations and visualizations.

## Results

### Isolation and identification of foodborne pathogens

In total, 54 pathogenic bacteria were isolated from 6 seafood samples (crab, pollack roe, seaweed fulvescens, octopus, mussel, and sea urchin). These pathogens were identified as *V. vulnificus* (1 strain), *V. parahaemolyticus* (1 strain), non-O1-type *V. cholerae* (1 strain), *Y. enterocolitica* (1 strain), *B. cereus* (1 strain), *S. aureus* (1 strain), *Pseudomonas aeruginosa* (16 strains), *E. coli* (8 strains), *Klebsiella pneumonia* (2 strains), *Enterococcus hirae* (9 strains), *Enterococcus faecalis* (3 strains), *Listeria innocua* (4 strains), and *Serratia liquefaciens* (6 strains) at the molecular level using 16S rRNA gene sequencing. Among these pathogens, six strains of *B. cereus*, *Y. enterocolitica*, *S. aureus, V. cholera, V. parahaemolyticus,* and *V. vulnificus* were selected as target pathogens, and three *S. aureus* type strains were also selected (Table 1) as these bacterial species have been associated previously with agricultural water contamination related to potential foodborne disease outbreaks (Pianetti et al., 2004; Silva et al., 2020; Elshikh et al., 2022; Roulová et al., 2022).

### General genome features of selected foodborne pathogens, and the design of primer sets

The genome sequence information of selected target pathogens is required to design specific primer sets and confirm their binding sites in the genomes. Therefore, NGS genome sequencing was performed, and draft genome sequences were obtained for *B. cereus*, *Y. enterocolitica*, *V. cholera, V. parahaemolyticus,* and *V. vulnificus* as well as two *S. aureus* strains (SG_001 and CCARM 3089). In addition, the previously reported genome sequences of two *S. aureus* strains (ATCC 23235 and Newman) were obtained from the NCBI GenBank database. The general genome features of these foodborne pathogens are summarized in Table 2. Based on the genome sequences, primer sets targeting specific toxin genes and virulence factors were designed to meet the criteria of primer design given in Materials and Methods. The sequence information of the designed primer sets is shown in Table 3, and the primer target genes and primer binding locations are listed in Supplementary Table S1.

**Table 2:** General genome features of foodborne pathogens.

Bacterium	Strain	Genome size (bp)	Assembly	Contig	GC (%)	CDS	tRNA	rRNA	Reference^d^
**Selected foodborne pathogens**									
*Bacillus cereus*	SG_003	5,908,983	Draft	83	34.81	5,920	62	3	This study
*Yersinia enterocolitica*	SG_002	4,357,829	Draft	123	46.92	3,932	66	3	This study
*Staphylococcus aureus*	ATCC 23235^a^	2,789,574	Draft	2	32.68	2,705	59	19	ATCC
	Newman^b^	2,878,897	Complete	1	32.89	2,851	59	16	ATCC
	CCARM 3089^c^	2,865,317	Draft	54	32.72	2,822	56	2	CCARM
	SG_001	2,944,975	Draft	111	32.77	2,781	59	4	This study
*Vibrio cholerae*	SG_017	4,005,842	Draft	91	47.52	3,592	69	4	This study
*Vibrio parahaemolyticus*	SG_014	6,040,036	Draft	81	44.01	5,740	135	5	This study
*Vibrio vulnificus*	SG_012	5,012,927	Draft	114	46.66	4,401	83	4	This study

a NCBI GenBank BioProject accession number, PRJNA224116.

b NCBI GenBank BioProject accession number, PRJDA18801.

c NCBI GenBank BioProject accession number, PRJNA870224.

d ATCC, American Type Culture Collection; CCARM, Culture Collection of Antimicrobial Resistance Microbes.

**Table 3:** Selected pathogen species-specific genes, their functions, and the associated designed primer sets.

Bacterium	Gene	Function	Primer	Sequence (5′ to 3′)	Size (bp)	Reference
*Bacillus cereus*	*entFM1*	Enterotoxin	ent_F	GAACTGCTGGTACAACACCTG	229	This study
			ent_R	TCTGCACTAATGAACTGACCG		
	*tpi*	Triose phosphate isomerase	tpi_F	GCGCTCTTCTAAAGTCTCAC	175	This study
			tpi_R	CGAAATTAGCCCAGTAGCAC		
*Yersinia enterocolitica*	*ail*	Attachment invasion locus protein	ail_F	TGGGGCCATCTTTCCGCATTA	235	This study
			ail_R	TACCCTGCACCAAGCATCCAA		
	*gspE*	Type II secretion system ATPase	gspE_F	AACGGGGCATCTGGTTCTCTC	190	This study
			gspE_R	TGGTGGTGTCAGGAAAGGGAC		
*Staphylococcus aureus*	*femA*	Methicillin resistance factor	femA_F	GCAGCTTGCTTACTTACTGCT	214	This study
			femA_R	TACCTGTAATCTCGCCATCAT		
	*sea1*	Exotoxin A	sea_F	ATTCATTGCCCTAACGTGGAC	191	This study
			sea_R	GCTGTAAAAATTGATCGTGACTCTC		
	*seb1*	Exotoxin B	seb_F	GTATGGTGGTGTAACTGAGC	212	This study
			seb_R	CCGTTTCATAAGGCGAGTTG		
	*sec1*	Exotoxin C	sec_F	CTGCTATTTTTCATCCAAAGA	180	This study
			sec_R	TTCTTATCAGTTTGCACTTCA		
	*sed1*	Exotoxin D	sed_F	TGTCACTCCACACGAAGGTA	162	This study
			sed_R	TGCAAATAGCGCCTTGCTTG		
*Vibrio cholerae*	*ctxA*	Enterotoxin	ctxA_F	GCCAAGAGGACAGAGTGAGTA	253	This study
			ctxA_R	ATGAGGACTGTATGCCCCTA		
	*hlyA*	Cytolysin and hemolysin	hlyA_F	GTTTGTATGTGCGAGCGGGTG	175	This study
			hlyA_R	GTGAATGTCAGCGCCACCAAC		
	*toxS*	Transmembrane regulator	toxS_F	TAAGACCAACAGCAACCGCCC	209	This study
			toxS_R	ACTCGACTGGCGTAACCAAAAGG		
*Vibrio parahaemolyticus*	*plsX*	Phosphate acyltransferase	plsX_F	GCACTGTCTCATTTCCCAGAG	219	This study
			plsX_R	CGCTTCTTGGTCAGAAACCAG		
	*tdh*	Thermostable direct hemolysin	tdh_F	TCCATCTGTCCCTTTTCCTGCC	187	This study
			tdh_R	CAGCCATTTAGTACCTGACGTTGTG		
	*tlh*	Thermolabile hemolysin precursor	tlh_F	GCGAGCGATCCTTGTTTGGAC	144	This study
			tlh_R	GCGGTGAGTTGCTGTTGTTGG		
	*toxR*	Transcriptional activator	toxR_F	ACCTGTGGCTTCTGCTGTG	178	This study
			toxR_R	CCAGTTGTTGATTTGCGGGTG		
*Vibrio vulnificus*	*glnA*	Glutamate ammonia ligase	glnA_F	AGCACATCTCTATTCCTTCTC	170	This study
			glnA_R	TAGCGTTGCTTCTTCAGTAA		
	*vvh*	Hemolysin	vvh_F	CTCTGCCTAGATGTTTATGG	199	This study
			vvh_R	CAATACCATTTCTGTGCTAAG		

### Singleplex PCR for NGS panel primer validation

**Figure 1:**
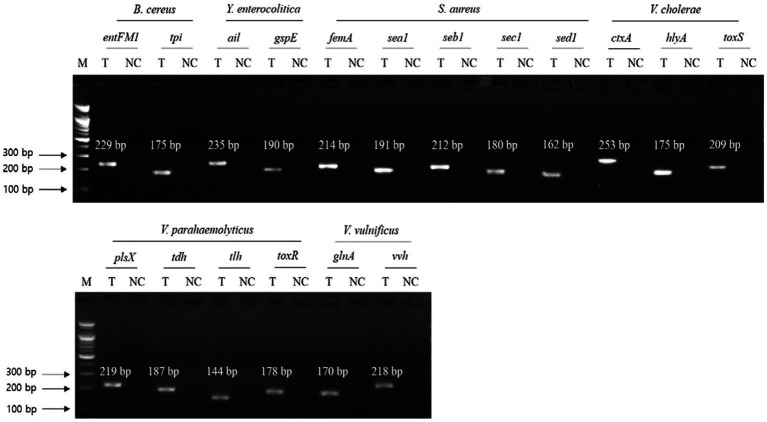
Gel electrophoresis results of singleplex PCR. Target pathogens and their specific genes are shown above the gel electrophoresis results. PCR mixture of the test (T) lane contained the associated target pathogen genomic DNA and specific gene primer set. PCR mixture of the negative control (NC) lane contained molecular water and the target pathogen-specific gene primer set. M: 100 bp DNA ladder.

To evaluate the specificity of primer sets to the target pathogens, singleplex PCR was performed with a single target pathogen and an associated single primer set. For the six target pathogens, the selected specific genes with their encoded functions, designed specific primer sets, and expected PCR amplicon sizes are listed in Table 3. Following singleplex PCR, agarose gel electrophoresis analysis revealed that all PCR amplicons were of the expected size according to the single PCR bands, confirming the specificity of all the PCR primer sets to the associated target pathogens (Figure 1). Thus, the designed primer sets qualified for crosscheck PCR evaluation in the next stage.

### Crosscheck PCR for NGS panel primer validation

To confirm the specificity of the primer sets to the target and nontarget pathogens, two different crosscheck PCRs were conducted: (1) an evaluation of the target pathogen and eight different nontarget pathogens with a single primer set and (2) an evaluation of a single pathogen-targeting primer set mixture (2–5 primer sets) with an associated target pathogen.

**Figure 2:**
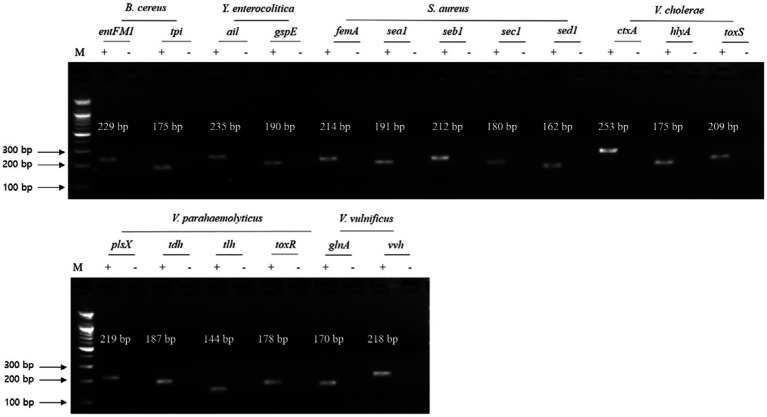
Gel electrophoresis results of the first crosscheck PCR. Target pathogens and their specific genes are shown above the gel electrophoresis results. PCR mixture of the test (+) lane contained a genomic DNA mixture including the associated target pathogen and target pathogen-specific gene primer set. PCR mixture of the negative test (−) lane contained a genomic DNA mixture lacking the associated target pathogen and target pathogens-specific gene primer set. M: 100 bp DNA ladder.

For the first crosscheck PCR, two genomic DNA template sets (test DNA template mixture and negative control DNA template mixture without target pathogenic DNA) were prepared to confirm the nonspecific binding of a selected single primer set to nontarget pathogenic DNA. In this crosscheck PCR, the PCR amplicon bands specific to the selected gene were found in the target pathogen but not the nontarget pathogens (Figure 2). In addition, the sizes of the PCR amplicon bands matched those expected, indicating that the primer sets were highly specific to the target pathogenic DNA, even though the template DNA mixture contained all other nontarget pathogenic DNA. Thus, the PCR primer sets were specific to the associated target gene as well as the target pathogen.

**Figure 3:**
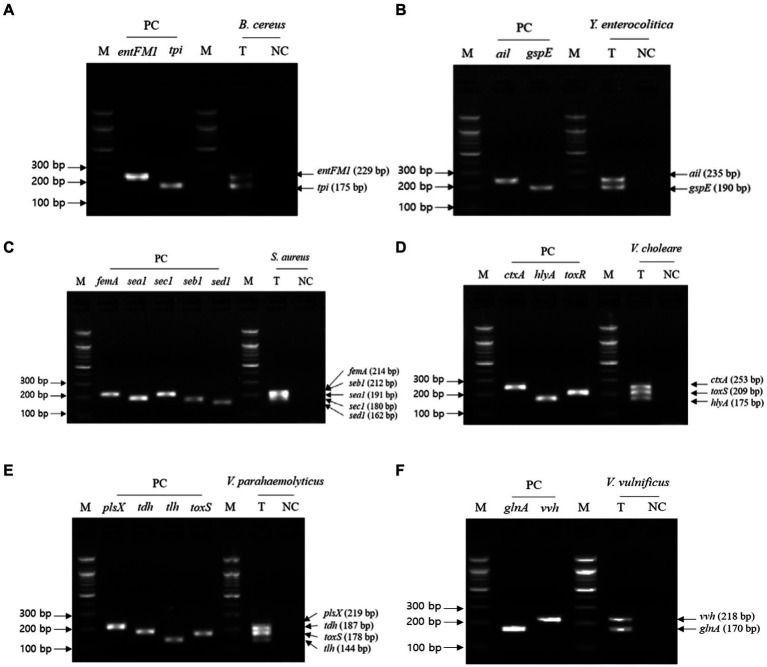
Gel electrophoresis results of the second crosscheck PCR. Target pathogens and their specific genes are shown above the gel electrophoresis results. The target pathogen-specific genes and their corresponding pathogens are as follows: **(A)** *B. cereus*, **(B)** *Y. enterocolitica*, **(C)** *S. aureus*, **(D)** *V. cholerae*, **(E)** *V. parahaemolyticus*, and **(F)** *V.vulnificus*. Lanes contain each target pathogen-specific gene with singleplex PCR amplicons as positive controls (PC). The PCR mixture of the test (T) lane contained the associated target pathogen genomic DNA and 2–5 target pathogen-specific gene primer sets. The PCR mixture of the negative control (NC) lane contained molecular water and 2–5 target pathogenspecific gene primer sets. M: 100 bp DNA ladder.

The second crosscheck PCR was conducted to determine whether one PCR reaction can multidetect the target genes in a single pathogen with a single pathogen-targeting primer set mixture that combines primer sets targeting 2–5 selected genes in a single pathogen (Table 3). In this crosscheck PCR, the PCR amplicons of all target genes in each pathogen were confirmed in the gel electrophoresis (Figure 3), and their amplicon sizes matched those expected. Therefore, PCR with a mixture of primer sets detected target genes in one reaction without any primer interference.

### Multiplex PCR for NGS panel primer validation

**Figure 4:**
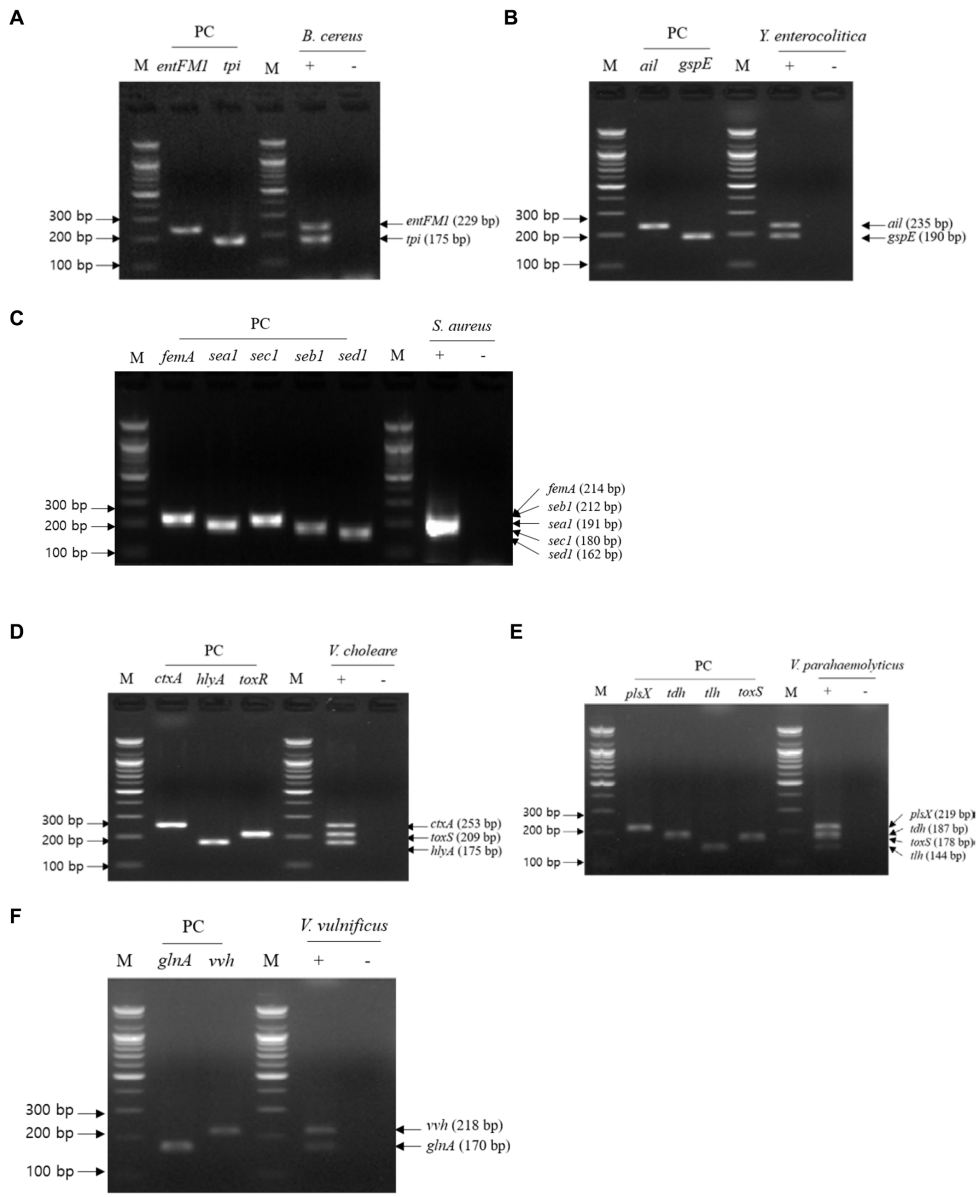
Gel electrophoresis results of multiplex PCR. Target pathogens and their specific genes are shown above the gel electrophoresis results. The target pathogen-specific genes and their corresponding pathogens are as follows: **(A)** *B. cereus*, **(B)** *Y. enterocolitica*, **(C)** *S. aureus*, **(D)** *V. cholerae*, **(E)** *V. parahaemolyticus*, and **(F)** *V.vulnificus*. Lanes contain each target pathogen-specific gene with singleplex PCR amplicons as positive controls. PCR mixture of the test (+) lane contained a genomic DNA mixture including the associated target pathogen and 2–5 target pathogen-specific gene primer sets. PCR mixture of the negative test (−) lane contained a genomic DNA mixture lacking the associated target pathogen and 2–5 target pathogens-specific gene primer sets. M: 100 bp DNA ladder.

For multiplex PCR, template DNA was prepared with the same sets used in the first crosscheck PCR, and the mixture of primer sets was the same as that used in the second crosscheck PCR. Multiplex PCR results showed that the PCR amplicons of all target genes in each pathogen were detected in gel electrophoresis, and their band sizes were the same as those expected (Figure 4). Therefore, multiplex PCR confirmed that a mixture of primer sets can be used to detect target genes in one reaction without any template DNA or primer interference.

### NGS panel analysis

**Figure 5:**
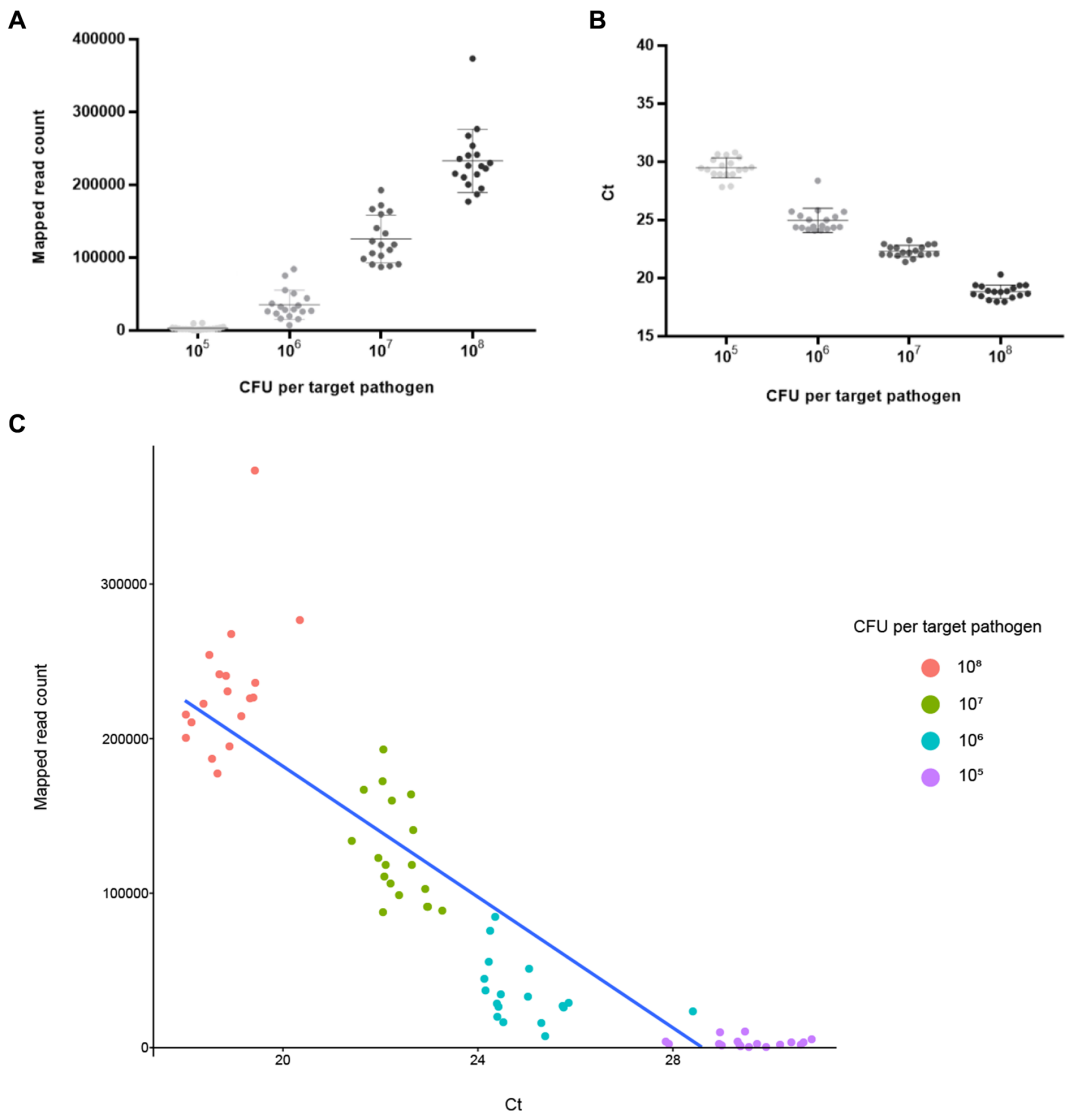
Analysis results of NGS panel and qPCR. Six different agricultural water samples with 10^8^, 10^7^, 10^6^, and 10^5^ CFUs per pathogen were analyzed using NGS panel analysis and qPCR. The analysis results are shown using linear regression with 10^8^, 10^7^, 10^6^, and 10^5^ CFUs per pathogen. Each point is the mean of six target pathogen-specific gene reads or Ct values in a single replicate. **(A)** Average reads mapped to the total target pathogen-specific genes (NGS panel). **(B)** Average total target pathogen Ct values (qPCR). **(C)** Correlation between the average reads mapped to the total target pathogen-specific genes and average total target pathogen Ct values.

NGS panel analysis was performed with six different agricultural water samples spiked with a mixture of target pathogens. Following NGS panel sequencing, the qualified sequence reads were collected and mapped to the target pathogen-specific gene sequences. The average number of sequence reads mapped to target pathogen-specific genes was 228,915 (93.263% of total qualified sequence reads), 125,902 (61.501%), 35,360 (23.125%), and 3,218 (1.879%) at dilutions of 10^8^, 10^7^, 10^6^, and 10^5^ CFUs per target pathogen, respectively. Interestingly, the averages number of sequence reads mapped to target pathogen-specific genes and the CFU number per target pathogen were positively associated ( y=78011x−95630 ; R^2^ = 0.9532; Figure 5A). The prepared negative control samples, which were not intentionally spiked with specific pathogen, exhibited 1–6 sequence reads mapped to target pathogen-specific genes, suggesting that a small number of pathogens were present in the original agricultural water samples and produced false positive results (Supplementary Figure S1A). Thus, ≤6 reads were determined as the false positive rate for further NGS panel analysis.

**Figure 6:**
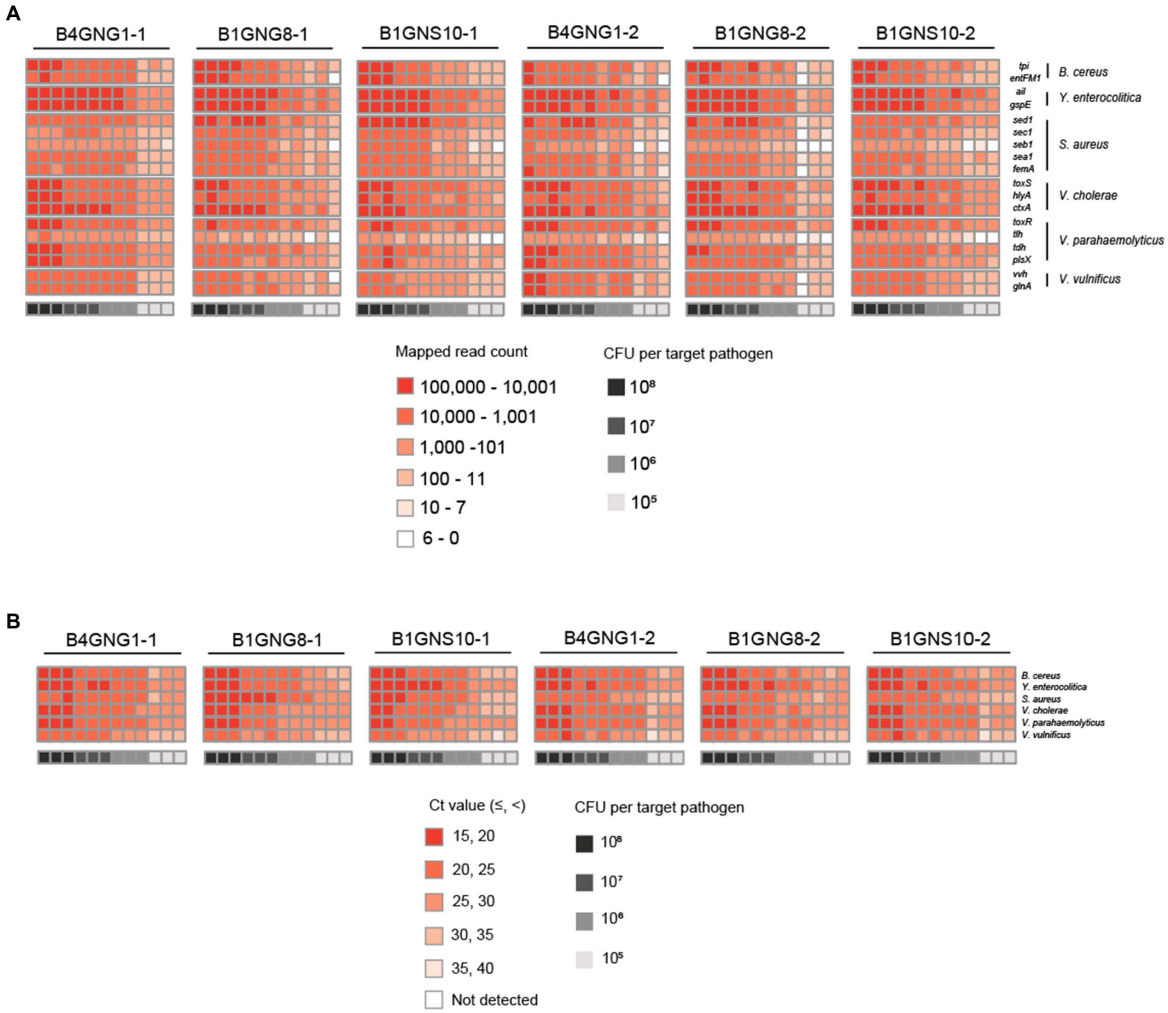
Heat maps of target pathogen detection results in six agricultural water samples with or without a mixture of target pathogens. **(A)** Target pathogen-specific gene reads of the NGS panel. **(B)** Target pathogen Ct values of qPCR. The color scale of the target pathogen-specific gene reads or target pathogen Ct values and the level of CFUs per target pathogen is indicated in the figure.

After mapping to 18 different target genes of six target pathogens, all qualified NGS panel sequence reads were collected from the six different agricultural water samples. The collected read counts for each dilution factor (10^8^, 10^7^, 10^6^, and 10^5^ CFUs per target pathogen) were compared in terms of the detection and identification of specific target pathogens (Figure 6A). For the dilution factors 10^6^ to 10^8^, all 18 target genes were multi-detected, and the dilutions were enough to identify the six target pathogens in one NGS panel analysis without false positives (Supplementary Figures S1B–D). This result was confirmed in triplicate tests of all agricultural water samples. As expected, the serial dilution of target pathogens was proportionally associated with the read count, i.e., the highest and lowest numbers of read counts were associated with the dilution factors 10^8^ and 10^6^, respectively, supporting the results shown in Figure 5A. However, when the dilution factor was 10^5^, many false positive results were detected (Supplementary Figure S1E). In particular, *tlh* of *V. parahaemolyticus* and *seb1* of *S. aureus* were poorly detected by NGS panel analysis. The number of read counts for each target gene was compared among dilution factors, and the numbers of *tlh* and *seb1* were always lower than those of the other 16 target genes, supporting the results shown in Supplementary Figure S2. Therefore, *tlh* and *seb1* could be removed as target genes to increase the limit of detection and improve the identification of specific target pathogens in NGS panel analysis. Although false positive results were found at 10^5^, using the average of triplicate tests in NGS panel analysis removed most of the false positive results at this dilution, thereby enhancing the detection and identification of all target pathogens (Supplementary Figure S1E). Nevertheless, the results of NGS panel analysis suggest that the limit of detection and identification of target pathogens may be at a dilution of 10^5^ CFUs.

### qPCR analysis

The qPCR template DNA used was the same as that used in the NGS panel analysis. According to the qPCR results, the average Ct values of the target pathogens were 18.84 (10^8^ CFUs per target pathogen), 22.33 (10^7^), 24.97 (10^6^), and 29.49 (10^5^). Interestingly, a negative correlation existed between Ct and the number of cells of the target pathogen ( y=−3.4584x+32.556 ; R^2^ = 0.9895), suggesting that target pathogens with a low Ct value or high number of cells could be rapidly detected and identified (Figure 5B). In contrast, the prepared negative control samples without the presence of specific pathogens showed no Ct values across all qPCR reactions (up to 40 cycles), suggesting that target pathogens were not present in these negative control samples (Supplementary Figure S3A).

Furthermore, the Ct values per target pathogen at four different dilution factors (10^8^, 10^7^, 10^6^, and 10^5^ CFUs per target pathogen) were compared to determine the sensitivity and detection limit of qPCR (Figure 6B). For all dilution factors, all target pathogens were detected in the qPCR reactions, and the six different target pathogens were identified without false positives (Supplementary Figures S3B–E). This result was confirmed in triplicate tests of all agricultural water samples. Overall, these results suggest that the sensitivity of qPCR may be higher than that of NGS panel analysis.

### Comparative evaluation of NGS panel analysis and qPCR

**Figure 7:**
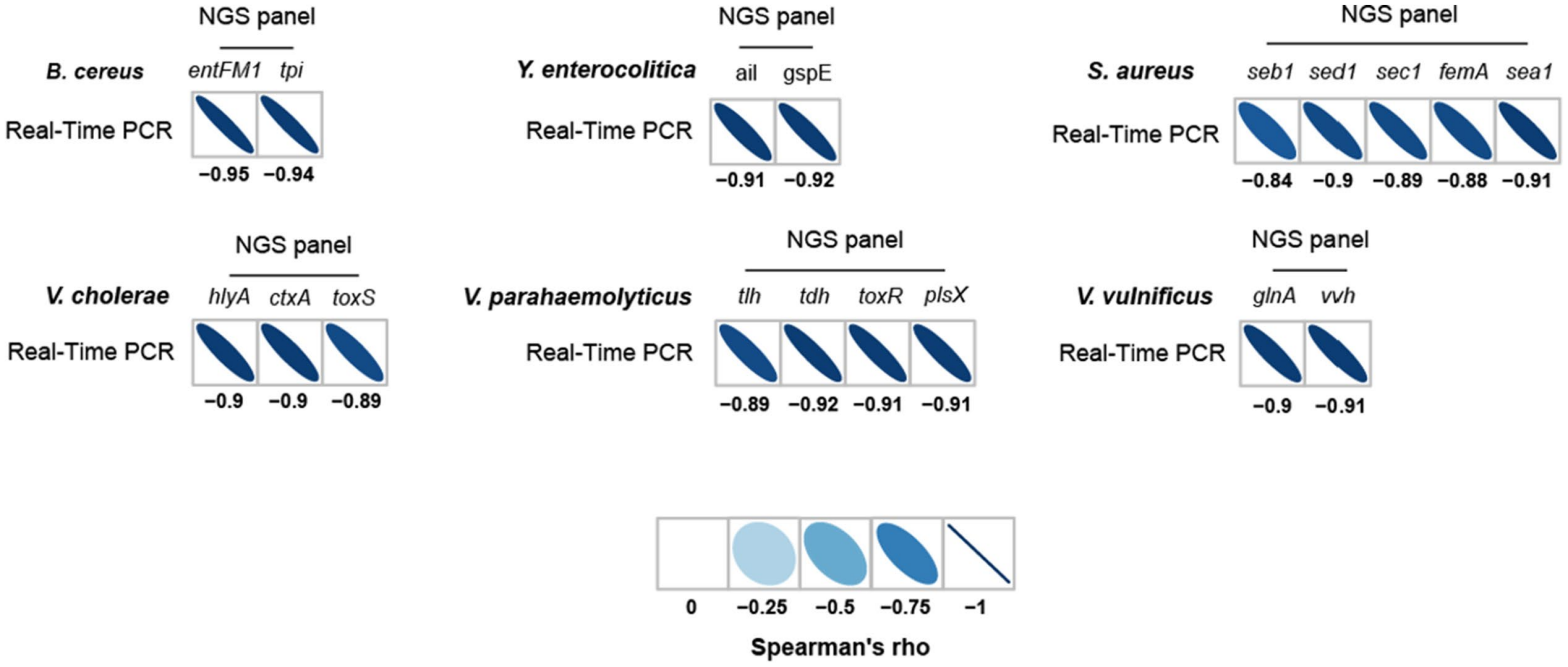
Spearman correlation between NGS panel analysis and multiplex qPCR results for six detected target pathogens.

To further evaluate the detection and identification of specific target pathogens using NGS panel analysis, the results of NGS panel analysis and qPCR were compared. As the qualified read counts and Ct values were correlated with the number of cells of the target pathogens, additional correlation analyses between the qualified read counts and Ct values in each specific target pathogen were performed. Interestingly, the read counts and Ct values were negatively correlated, with comparative analysis revealing a negative relationship ( y=−21154x+605174 ; R^2^ = 0.7984; Figure 5C), supporting the previous finding that a high number of target pathogen cells is associated with the quicker detection and identification of pathogens. To verify this correlation, Spearman correlation analysis was performed using the results of NGS panel analysis and qPCR for specific target genes, and negative correlations were found in all cases (Figure 7). The genes *entFM1* and *tpi* of *B. cereus* exhibited the highest correlations, whereas *seb1* of *S. aureus* exhibited the lowest correlation (Figure 7), which might have been due to the false negative results for this gene in NGS panel analysis (Supplementary Figure S1E).

The strong correlation between NGS panel analysis and qPCR in the specific target genes of pathogens suggests that the newly developed NGS panel analysis could serve as a supporting or alternative method to qPCR for the detection and identification of multiple target pathogens in given environments.

## Discussion

Foodborne disease outbreaks are generally associated with foodborne pathogen contamination (Newell et al., 2010). However, food environments contain a plethora of food-related microbiota including a variety foodborne pathogens; (Lampel et al., 2000) therefore, it is often a challenge to detect and identify the specific foodborne pathogen that has caused an outbreak. To detect and identify outbreak-causing pathogens, various methods can be used such as culturing with specific selective media, immune detection using specific antibodies, PCR using foodborne pathogen-targeting specific primer sets, and real-time PCR. However, these methods are not always appropriate for detecting and identifying specific pathogens in foodborne disease outbreaks owing to the complex bacterial ecosystem of food environments. Therefore, new screening methods must be developed and optimized. Accordingly, in the present study, a new method for detecting and identifying multiple pathogens in one reaction, an NGS panel method, was developed, optimized, and compared with a typical qPCR method.

Although the NGS panel method was originally developed for clinical diagnosis and GMO detection (Arulandhu et al., 2018; López-Reig et al., 2019) it has also been used for the multiple detection and identification of foodborne pathogens in food samples (Ferrario et al., 2017). In the current study, NGS panel primer sets targeting 18 pathogenic genes were developed and optimized. Using these primer sets, NGS panel analysis was conducted using six agricultural water samples spiked with pathogens. All pathogens were detected and identified, even with a sample dilution of 10^5^ CFUs per pathogen, demonstrating the main advantage of the new method: multiple pathogen detection in one reaction. However, comparative analysis revealed that qPCR has a higher sensitivity than the NGS panel method, although all pathogens could not be detected in one reaction using qPCR. The NGS panel method also gave some false positive results when the number of target pathogen cells was low. Thus, the sensitivity of the NGS panel method when detecting and identifying pathogens must be increased through further optimization of the primer sets. Another major disadvantage of the NGS panel method is the time required to complete NGS sequencing, which could also be optimized through the use of new NGS sequencing technology. For example, nanopore sequencing technology can achieve real-time sequencing (Buytaers et al., 2021) and would therefore be a candidate sequencing method for minimizing the sequencing time in NGS panel analysis. In summary, although the potential and advantages of the developed NGS panel analysis method were demonstrated in this study, further optimization of the NGS panel primer sets and the application of new real-time NGS sequencing technology will enhance the method’s pathogen detection and identification capabilities and help popularize the technology for the improvement of food safety.

## Conclusion

Because general methods cannot multi-detect and identify complex foodborne pathogens of a food sample in a reaction with high sensitivity, the NGS panel analysis method was optimized and its performance was evaluated with multiple detection of 18 virulence factor genes of 6 selected foodborne pathogens at the same time up to dilution factor of 10^5^ in this study. Although this method overcomes the limitation of other detection methods in multiple detection and identification, its comparative analysis with qPCR showed that NGS panel analysis has lower sensitivity and longer detection/identification time than qPCR due to NGS sequencing. However, qPCR could detect and identify only a few pathogenic bacteria in a reaction. To improve the sensitivity and detection/identification time of NGS panel analysis, the primer sets need to be further optimized and a new real-time sequencing technology such as nanopore sequencing should be used in the next study. The optimized primer sets and the real-time nanopore sequencing technology would reduce the false-positive results as well as the sequencing time in NGS panel analysis. Although NGS panel analysis method still needs some improvements, this new technology enabled to multi-detect and identify numerous foodborne pathogens at the same time by a reaction, and even it would be able to determine the origin pathogen in foodborne outbreaks. Therefore, this study provides information on the usability and application of NGS panel analysis method. Furthermore, based on this, introduction of further optimized new NGS panel primer sets and nanopore sequencing technology in the next study will make possible the real-time detection and identification of pathogens during NGS sequencing as well as rapid determination of the origin pathogen from foodborne outbreaks.

## Data availability statement

The datasets presented in this study can be found in online repositories. The genome sequences of six foodborne pathogenic bacteria listed in Table 2 were deposited in the GenBank database with the BioProject accession numbers PRJNA882507 (*S. aureus* SG_001) and PRJNA857825 (other pathogenic bacteria).

## Author contributions

JP, SoK, and J-HL: conceptualization. D-GP, E-SH, and J-HL: methodology. D-GP, J-GK, E-SH, IC, JC, WL, SeK, SoK, and J-HL: validation. D-GP and E-SH: formal analysis. BK: software. D-GP, E-SH, BK, WL, SeK, and SoK: investigation. D-GP, E-SH, BK, and IC: data curation. WL, SeK, and SoK: resources. D-GP, J-GK, and J-HL: writing-original draft. J-GK and J-HL: writing-review and editing. D-GP: visualization. JC, JP, and J-HL: supervision. J-HL: project administration and funding acquisition. All authors contributed to the article and approved the submitted version.

## Funding

This research was supported by a grant (20161MFDS030 and 21162MFDS027) from the Ministry of Food and Drug Safety in 2022 and by Cooperative Research Program for Agriculture Science and Technology Development (project no. PJ016298), Rural Development Administration, Republic of Korea.

## Conflict of interest

E-SH, BK, IC, JC, and JP were employed by Sanigen Co., Ltd.

The remaining authors declare that the research was conducted in the absence of any commercial or financial relationships that could be construed as a potential conflict of interest.

## Publisher’s note

All claims expressed in this article are solely those of the authors and do not necessarily represent those of their affiliated organizations, or those of the publisher, the editors and the reviewers. Any product that may be evaluated in this article, or claim that may be made by its manufacturer, is not guaranteed or endorsed by the publisher.

## Footnotes

^1^ https://www.ncbi.nlm.nih.gov/genbank
