# Supplementary material for: Novel next generation sequencing panel method for the multiple detection and identification of foodborne pathogens in agricultural wastewater
Source: Front Microbiol. 2023 Jul 13;14:1179934. doi: 10.3389/fmicb.2023.1179934 (PMC10374199; doi:10.3389/fmicb.2023.1179934)

**Supporting Information for**

**Novel Next Generation Sequencing Panel Method for the Multiple Detection and Identification of Foodborne Pathogens in Agricultural Wastewater**

**Dong-Geun Park^1,†^, Joon-Gi Kwon^1,†^, Eun-Su Ha^2^, Byungcheol Kang^2^, Iseul Choi^2^, Jeong-Eun Kwak^1^, Jinho Choi^2^, Woojung Lee^3^, Seung Hwan Kim^3^, Soon Han Kim^3^, Jeongwoong Park^2^** **and Ju-Hoon Lee^1*^**

*^1^ Department of Food and Animal Biotechnology, Department of Agricultural Biotechnology, Research Institute of Agriculture and Life Sciences, Center for Food and Bioconvergence, Seoul National University, Seoul 08826, Korea*

*^2^ Research and Development Center, Sanigen Co., Ltd, Anyang 14059, Korea*

*^3^ Division of Food Microbiology, National Institute of Food and Drug Safety Evaluation, Ministry of Food and Drug Safety, Cheongju 28159, Korea*

**^†^ These authors have contributed equally to this work and share first authorship**

***Corresponding author: Dr. Ju-Hoon Lee (juhlee@snu.ac.kr)**

**Table S1. Primer binding sites of selected pathogens-specific genes**

| Bacterium | Strain | Gene | Contig | Primer binding site | |
| --- | --- | --- | --- | --- | --- |
|  |  |  |  | Forward (nt) | Reverse (nt) |
| Selected foodborne pathogens |  |  |  |  |  |
| *Bacillus cereus* | SG_003 | *tpi* | Contig 4 | 148,119-148,138 | 147,964-147,983 |
|  |  | *entFM1* | Contig 12 | 62,720-62,740 | 62,512-62,532 |
| *Yersinia enterocolitica* | SG_002 | *ail* | Contig 62 | 1,310-1,330 | 1,524-1,544 |
|  |  | *gspE* | Contig 2 | 106,645-106,665 | 106,476-106,496 |
| *Staphylococcus aureus* | ATCC 23235 | *femA* | Contig 1 | 1,365,336-1,365,356 | 1,365,529-1,365,549 |
|  |  | *sed1* | Contig 2 | 27,108-27,127 | 26,966-26,958 |
|  | Newman | *femA* | Contig 1 | 1,417,190-1,417,210 | 1,417,383-1,417,403 |
|  |  | *sea1* | Contig 1 | 2,094,921-2,094,941 | 2,094,751-2,094,775 |
|  | CCARM 3089 | *femA* | Contig 10 | 15,889-15,919 | 15,706-15,726 |
|  |  | *sec1* | Contig 19 | 581-601 | 740-760 |
|  | SG_001 | *femA* | Contig 6 | 15,907-15,927 | 16,100-16,120 |
|  |  | *sea1* | Contig 39 | 410-430 | 576-600 |
|  |  | *seb1* | Contig 5 | 133,856-133,875 | 133,664-133,683 |
| *Vibrio cholerae* | SG_017 | *ctxA* | Contig 50 | 1,263-1,283 | 1,031-1,050 |
|  |  | *hlyA* | Contig 18 | 52,696-52,716 | 52,542-52,562 |
|  |  | *toxS* | Contig 5 | 110,538-110,558 | 110,350-110,372 |
| *parahaemolyticus* | SG_014 | *plsX* | Contig 6 | 263,592-263,612 | 263,790-263,810 |
|  |  | *tdh* | Contig 61 | 541-561 | 373-397 |
|  |  | *tlh* | Contig 1 | 92,117-92,137 | 91,994-92,104 |
|  |  | *toxR* | Contig 13 | 142,352-142,370 | 142,509-142,529 |
| *vulnificus* | SG_012 | *glnA* | Contig 18 | 28,556-28,568 | 28,417-28,436 |
|  |  | *vvh* | Contig 11 | 137,880-137,899 | 137,682-137,702 |

**Table S2. Summary of NGS panel outputs in six agricultural water samples with or without target pathogen**

| Sample | Replicate | CFU | Yield  (bp) | Raw read | Filtered read | Merged  read | Mapped read to  total target pathogens  specific genes |
| --- | --- | --- | --- | --- | --- | --- | --- |
| B4GNG1-1 | 1 | 10^8^ | 122,323,563 | 813,624 | 777,011 | 388,092 | 225,870 |
|  | 2 |  | 120,135,561 | 798,876 | 763,566 | 382,365 | 226,278 |
|  | 3 |  | 113,298,422 | 753,494 | 718,909 | 360,664 | 210,396 |
|  | 1 | 10^7^ | 102,606,782 | 681,612 | 651,757 | 324,788 | 117,955 |
|  | 2 |  | 102,524,845 | 681,180 | 650,391 | 322,964 | 133,514 |
|  | 3 |  | 88,639,999 | 589,026 | 564,287 | 283,870 | 87,610 |
|  | 1 | 10^6^ | 106,108,444 | 705,190 | 669,155 | 323,990 | 84,502 |
|  | 2 |  | 103,007,095 | 684,696 | 649,023 | 310,960 | 75,390 |
|  | 3 |  | 86,276,123 | 573,070 | 547,053 | 269,964 | 44,552 |
|  | 1 | 10^5^ | 72,610,739 | 483,468 | 443,534 | 226,855 | 2,094 |
|  | 2 |  | 77,685,911 | 515,642 | 490,066 | 244,924 | 3,782 |
|  | 3 |  | 80,914,390 | 536,924 | 515,769 | 260,710 | 2,229 |
|  | 1 | N.C. | 66,054,304 | 438,868 | 424,824 | 190,947 | 0 |
|  | 2 |  | 65,853,979 | 437,844 | 423,527 | 211,562 | 0 |
|  | 3 |  | 59,185,593 | 393,074 | 379,788 | 211,568 | 0 |
| B1GNG8-1 | 1 | 10^8^ | 122,323,563 | 813,624 | 687,548 | 343,648 | 215,557 |
|  | 2 |  | 113,840,513 | 756,878 | 724,938 | 362,091 | 214,609 |
|  | 3 |  | 93,555,990 | 622,006 | 595,322 | 299,075 | 200,636 |
|  | 1 | 10^7^ | 99,330,855 | 660,706 | 634,146 | 317,345 | 140,911 |
|  | 2 |  | 74,456,985 | 494,550 | 474,175 | 238,829 | 106,379 |
|  | 3 |  | 99,520,131 | 661,242 | 634,726 | 319,419 | 166,740 |
|  | 1 | 10^6^ | 75,483,859 | 501,438 | 480,227 | 241,545 | 51,230 |
|  | 2 |  | 68,273,889 | 452,794 | 434,637 | 219,744 | 7,581 |
|  | 3 |  | 73,256,917 | 486,380 | 467,071 | 236,470 | 26,051 |
|  | 1 | 10^5^ | 63,445,162 | 420,648 | 399,868 | 204,908 | 2,494 |
|  | 2 |  | 61,515,362 | 408,040 | 381,517 | 195,097 | 9,668 |
|  | 3 |  | 58,458,568 | 387,580 | 365,488 | 188,214 | 542 |
|  | 1 | N.C. | 65,557,295 | 435,878 | 420,622 | 197,030 | 0 |
|  | 2 |  | 59,703,457 | 396,256 | 382,110 | 193,337 | 1 |
|  | 3 |  | 62,023,488 | 412,520 | 397,587 | 209,078 | 1 |
| B1GNS10-1 | 1 | 10^8^ | 123,664,901 | 821,608 | 784,061 | 392,846 | 241,428 |
|  | 2 |  | 116,073,213 | 772,092 | 738,043 | 366,528 | 222,473 |
|  | 3 |  | 124,383,663 | 826,410 | 790,131 | 397,656 | 276,753 |
|  | 1 | 10^7^ | 84,073,800 | 558,692 | 532,378 | 256,122 | 98,591 |
|  | 2 |  | 87,042,497 | 579,368 | 551,385 | 265,964 | 91,250 |
|  | 3 |  | 91,044,828 | 605,604 | 576,959 | 280,197 | 102,806 |
|  | 1 | 10^6^ | 56,787,933 | 377,830 | 358,598 | 169,092 | 19,700 |
|  | 2 |  | 77,314,380 | 514,522 | 487,870 | 226,016 | 27,043 |
|  | 3 |  | 68,665,175 | 456,572 | 435,296 | 210,969 | 23,631 |
|  | 1 | 10^5^ | 67,997,614 | 451,950 | 429,353 | 210,247 | 3,555 |
|  | 2 |  | 60,372,011 | 401,234 | 380,691 | 181,631 | 3,168 |
|  | 3 |  | 62,857,231 | 417,616 | 398,239 | 195,931 | 1,814 |
|  | 1 | N.C. | 60,297,197 | 399,994 | 386,674 | 219,579 | 0 |
|  | 2 |  | 65,545,913 | 434,856 | 420,071 | 212,730 | 1 |
|  | 3 |  | 67,530,809 | 447,866 | 432,325 | 210,571 | 41 |
| B4GNG1-2 | 1 | 10^8^ | 128,060,503 | 851,038 | 816,231 | 414,953 | 373,558 |
|  | 2 |  | 109,095,267 | 725,606 | 695,131 | 349,362 | 267,587 |
|  | 3 |  | 103,081,871 | 685,992 | 655,534 | 323,582 | 177,227 |
|  | 1 | 10^7^ | 115,536,552 | 768,416 | 734,298 | 360,866 | 159,902 |
|  | 2 |  | 86,686,038 | 576,230 | 547,995 | 274,234 | 90,922 |
|  | 3 |  | 96,405,761 | 640,996 | 610,292 | 296,217 | 118,135 |
|  | 1 | 10^6^ | 62,068,027 | 413,482 | 391,195 | 185,051 | 26,659 |
|  | 2 |  | 87,005,474 | 578,864 | 550,731 | 263,528 | 55,599 |
|  | 3 |  | 71,146,388 | 473,260 | 446,615 | 206,356 | 37,194 |
|  | 1 | 10^5^ | 63,055,893 | 419,902 | 396,220 | 197,320 | 2,051 |
|  | 2 |  | 75,126,048 | 499,996 | 473,196 | 222,902 | 10,371 |
|  | 3 |  | 56,503,643 | 375,320 | 356,592 | 178,211 | 798 |
|  | 1 | N.C. | 71,908,602 | 477,388 | 462,875 | 219,006 | 0 |
|  | 2 |  | 60,639,953 | 402,194 | 387,715 | 197,508 | 0 |
|  | 3 |  | 52,633,882 | 349,186 | 335,987 | 234,027 | 41 |
| B1GNG8-2 | 1 | 10^8^ | 120,562,993 | 801,254 | 765,999 | 383,366 | 240,349 |
|  | 2 |  | 132,374,674 | 880,360 | 842,857 | 419,542 | 253,865 |
|  | 3 |  | 101,512,881 | 675,110 | 645,270 | 322,575 | 195,132 |
|  | 1 | 10^7^ | 99,860,057 | 663,998 | 637,040 | 320,456 | 122,916 |
|  | 2 |  | 91,555,312 | 608,690 | 583,612 | 292,394 | 110,905 |
|  | 3 |  | 123,499,309 | 820,032 | 787,067 | 394,779 | 193,057 |
|  | 1 | 10^6^ | 69,778,141 | 463,358 | 441,580 | 223,764 | 29,109 |
|  | 2 |  | 73,660,615 | 489,212 | 469,644 | 237,799 | 32,919 |
|  | 3 |  | 66,238,543 | 440,072 | 420,973 | 214,028 | 16,037 |
|  | 1 | 10^5^ | 72,560,042 | 480,974 | 461,927 | 236,193 | 309 |
|  | 2 |  | 68,935,920 | 457,018 | 436,178 | 223,234 | 1,879 |
|  | 3 |  | 73,524,495 | 488,064 | 467,809 | 239,006 | 1,574 |
|  | 1 | N.C. | 62,653,766 | 416,168 | 401,228 | 185,585 | 0 |
|  | 2 |  | 59,709,934 | 396,218 | 381,320 | 193,092 | 0 |
|  | 3 |  | 58,326,877 | 387,474 | 374,416 | 199,921 | 0 |
| B1GNS10-2 | 1 | 10^8^ | 112,438,475 | 748,812 | 718,785 | 360,441 | 235,971 |
|  | 2 |  | 121,976,971 | 811,692 | 777,439 | 388,050 | 230,360 |
|  | 3 |  | 101,291,935 | 674,346 | 645,012 | 321,367 | 187,065 |
|  | 1 | 10^7^ | 104,488,255 | 695,116 | 666,547 | 332,557 | 163,697 |
|  | 2 |  | 92,995,760 | 618,334 | 592,178 | 295,550 | 88,708 |
|  | 3 |  | 115,184,019 | 766,150 | 733,282 | 363,411 | 172,234 |
|  | 1 | 10^6^ | 77,869,355 | 518,622 | 492,483 | 236,345 | 28,517 |
|  | 2 |  | 71,894,289 | 478,292 | 459,399 | 228,707 | 16,467 |
|  | 3 |  | 76,260,756 | 507,146 | 485,085 | 238,776 | 34,297 |
|  | 1 | 10^5^ | 76,824,446 | 511,034 | 484,205 | 232,124 | 5,208 |
|  | 2 |  | 80,309,982 | 535,220 | 508,834 | 240,929 | 3,845 |
|  | 3 |  | 70,496,175 | 468,728 | 447,213 | 218,982 | 2,538 |
|  | 1 | N.C. | 63,724,702 | 423,400 | 409,258 | 176,867 | 1 |
|  | 2 |  | 62,410,362 | 414,276 | 400,895 | 201,315 | 0 |
|  | 3 |  | 52,801,398 | 350,818 | 338,890 | 204,450 | 0 |

**Supplementary figure legends**

**Figure S1.** Target pathogens-specific genes mapped read in six agricultural water samples with target pathogens using NGS panel. (A) Six agricultural water samples without target pathogen, (B) Six agricultural water samples with 10^8^ CFU per target pathogen, (C) Six agricultural water samples with 10^7^ CFU per target pathogen, (D) Six agricultural water samples with 10^6^ CFU per target pathogen, and (E) Six agricultural water samples with 10^5^ CFU per target pathogen. Agricultural water sample name and replicate numbers, target pathogens-specific genes and target pathogens, and read color scales were indicated on left, down, and right side of the figure, respectively.

**Figure S2.** Average read summary of target pathogens-specific genes of NGS panel analysis result in six agricultural water samples with target pathogens. Mapped read counts to target pathogens-specific genes in 10^8^, 10^7^, 10^6^, and 10^5^ CFU per target pathogens were indicated in figure as red, green, blue, and purple, respectively, and dashed line indicates total average read in each CFU per target pathogens. Upper error bar means maximum read counts and lower error bar means minimum read counts.

**Figure S3.** Target pathogens Ct-value in six agricultural water samples with or without target pathogens using qPCR. (A) Six agricultural water samples without target pathogen, (B) Six agricultural water samples with 10^8^ CFU per target pathogen, (C) Six agricultural water samples with 10^7^ CFU per target pathogen, (D) Six agricultural water samples with 10^6^ CFU per target pathogen, and (E) Six agricultural water samples with 10^5^ CFU per target pathogen. Agricultural water sample names and replicate numbers, target pathogens, and Ct-value color scales were indicated on left, down, and right side of the figure, respectively and N.D. indicates Ct values were not exceeded threshold until 40 cycles.

**Fig. S1.**

**A**


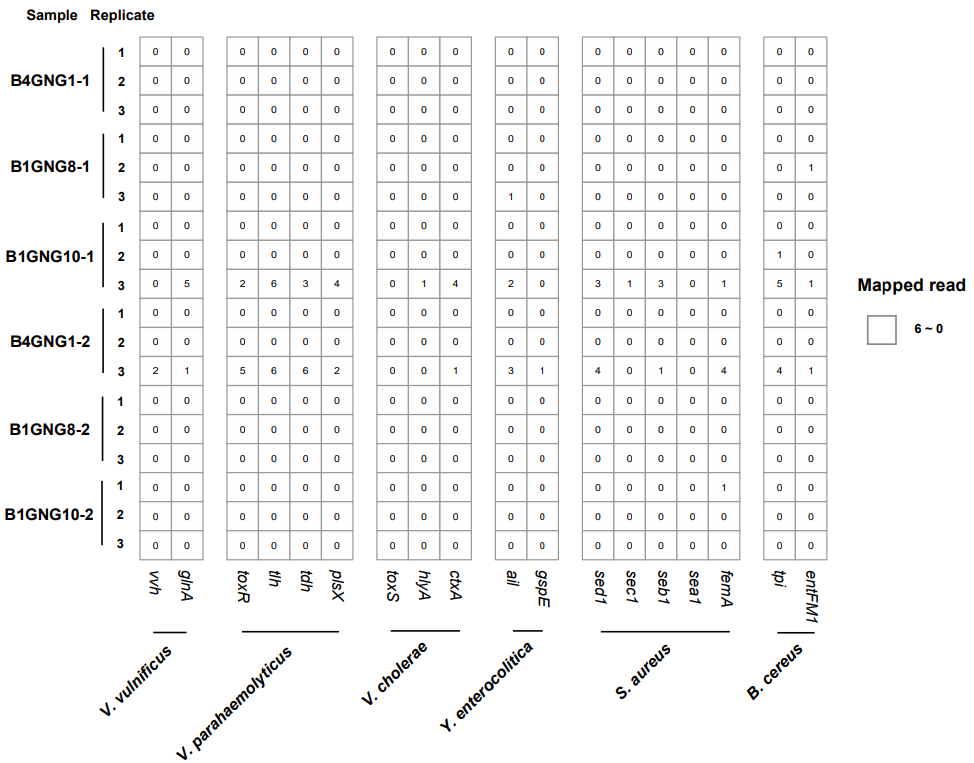


**Fig. S1. Cont.**

**B**


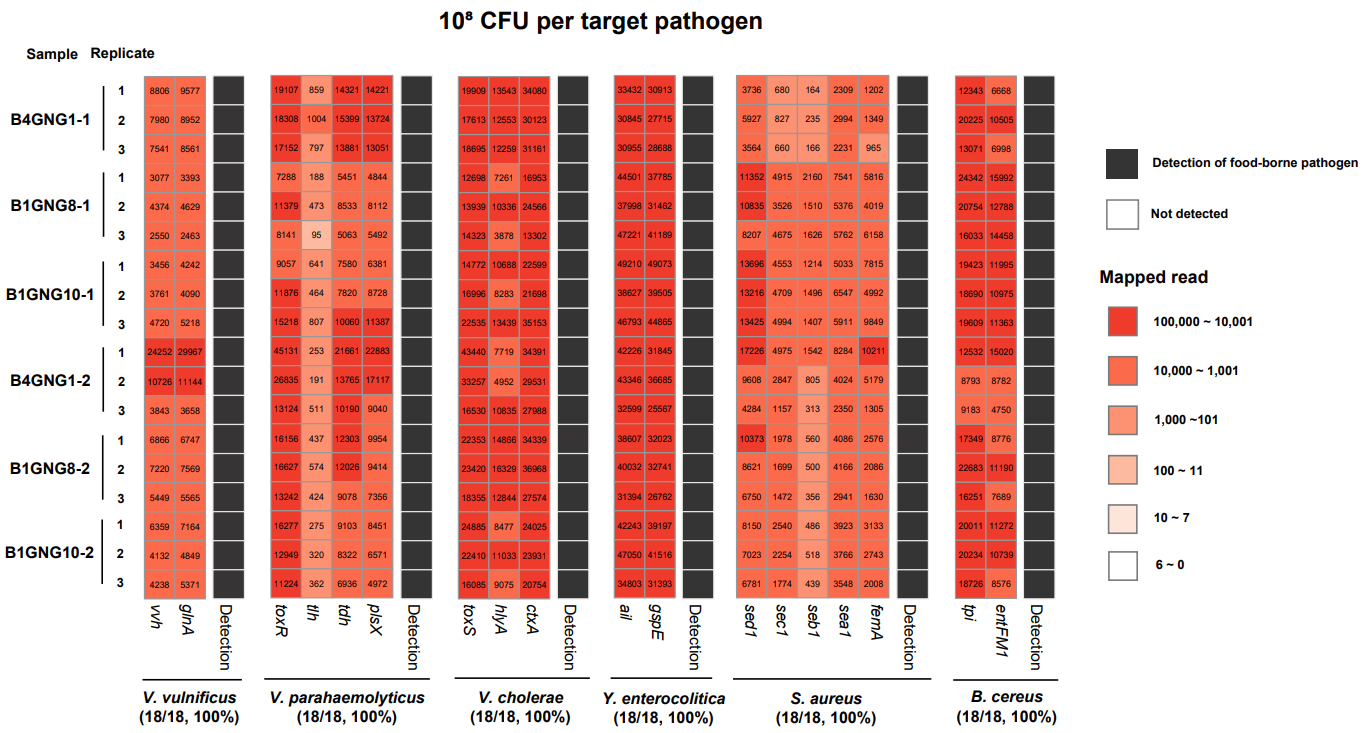


**Fig. S1. Cont.**

**C**


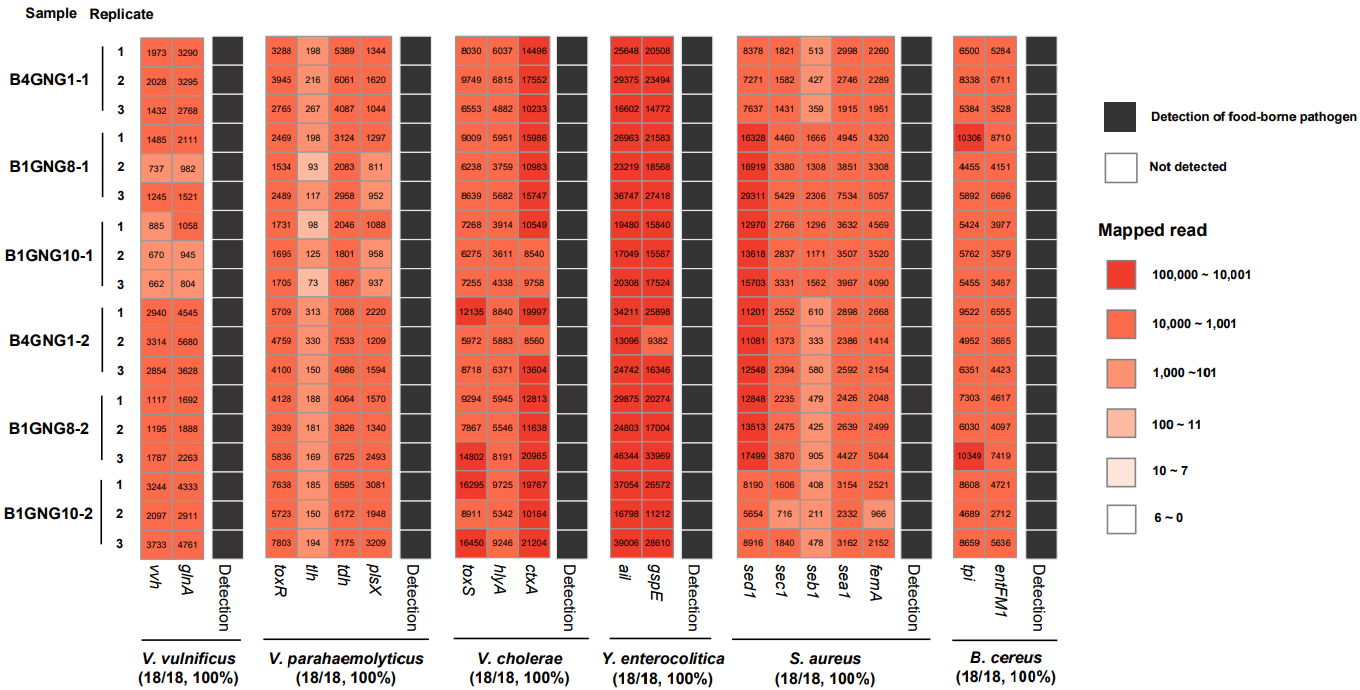


**Fig. S1. Cont.**

**D**


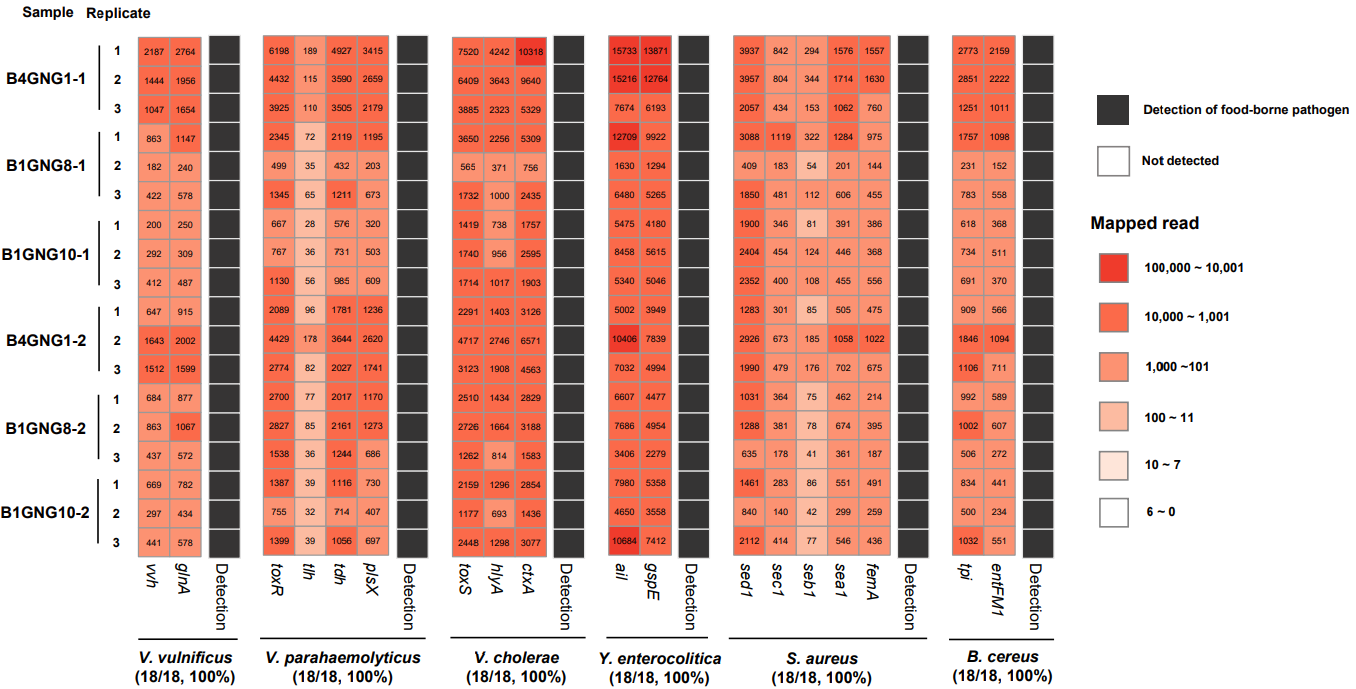


**Fig S1. Cont.**

**E**


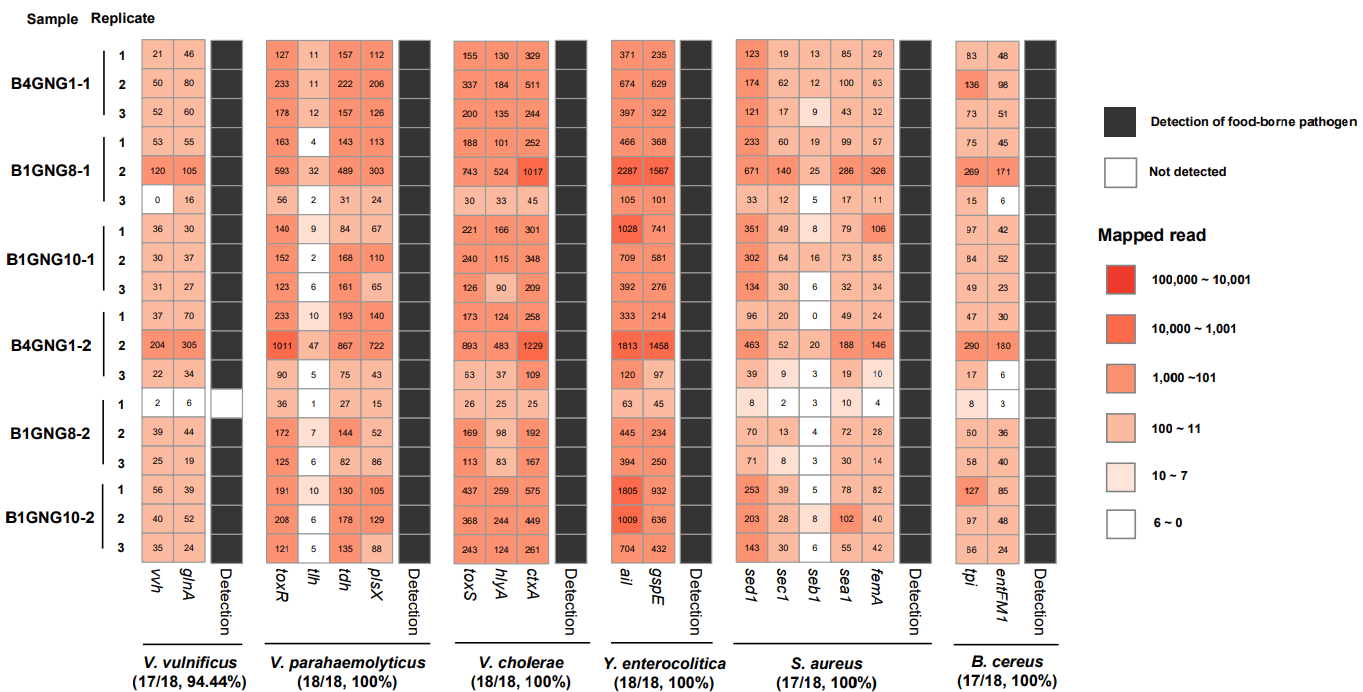


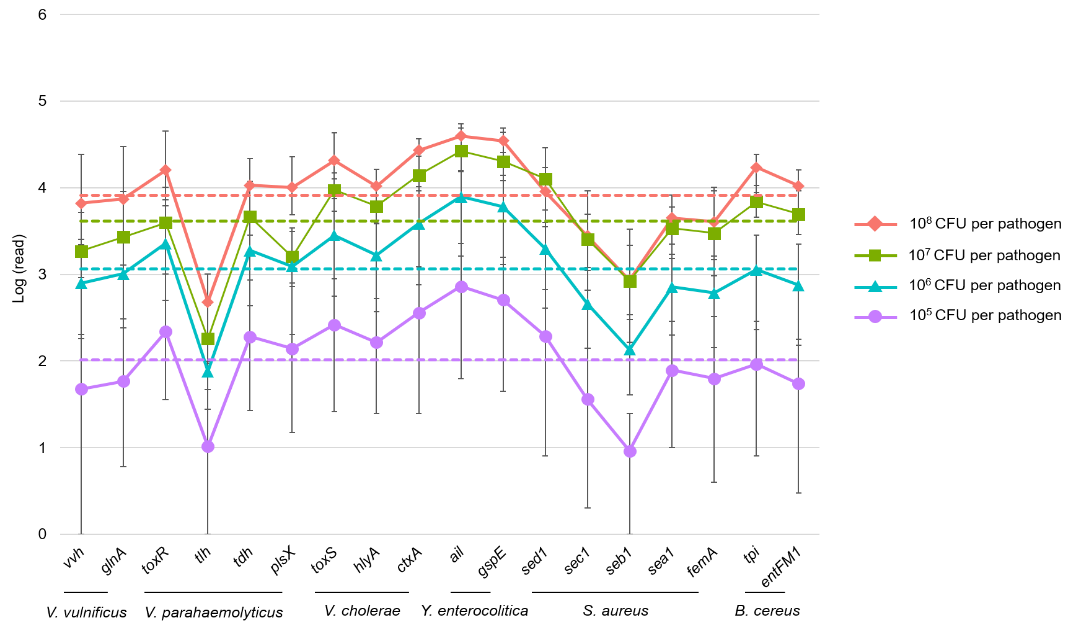
**Fig. S2.**

**Fig. S3.**

**A B**


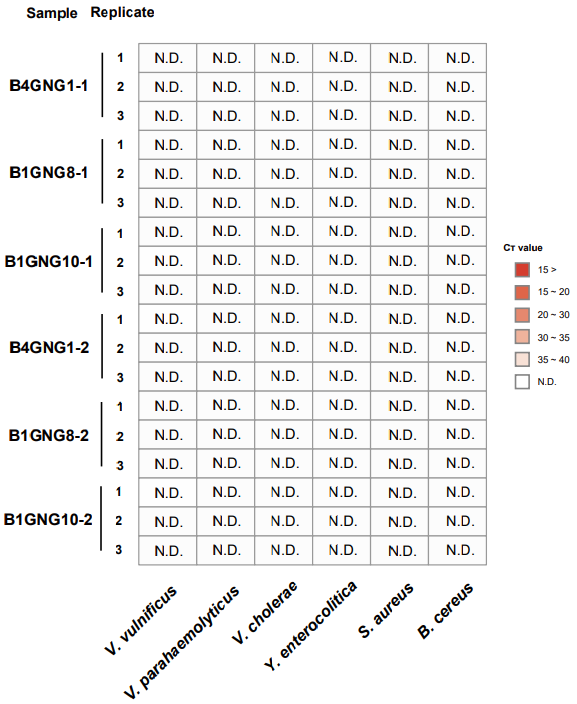

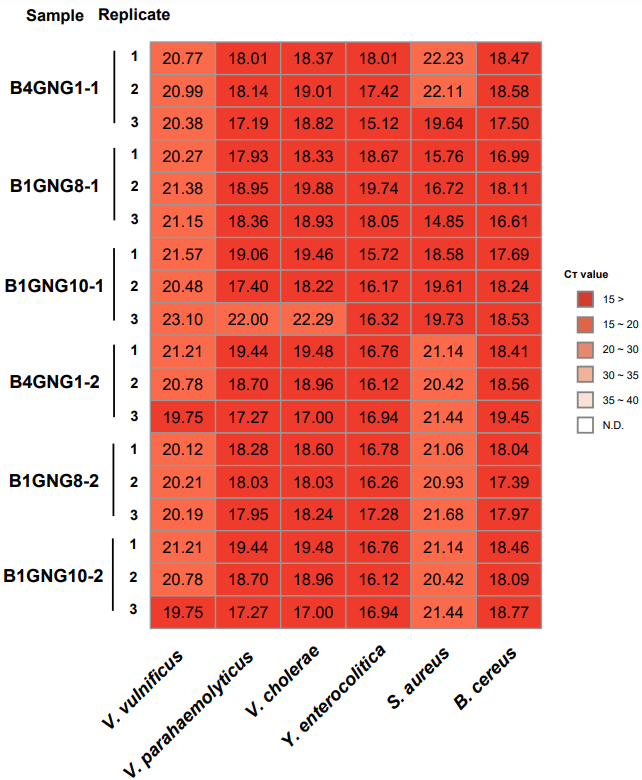


**Fig. S3. Cont.**

**C D**


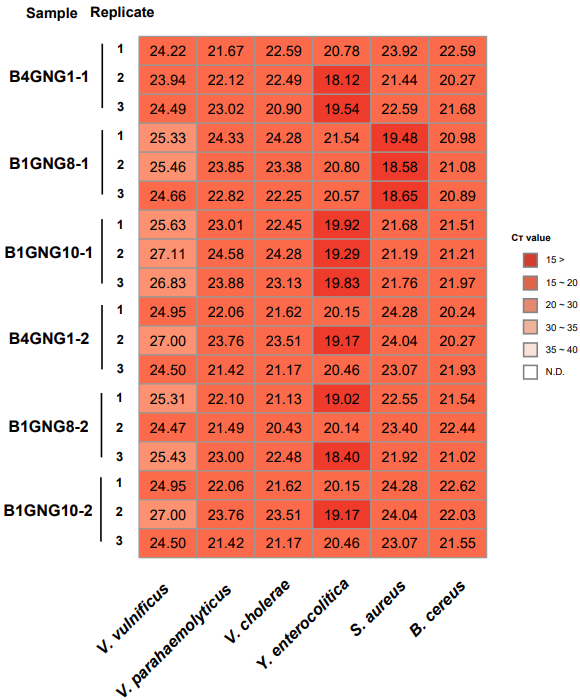

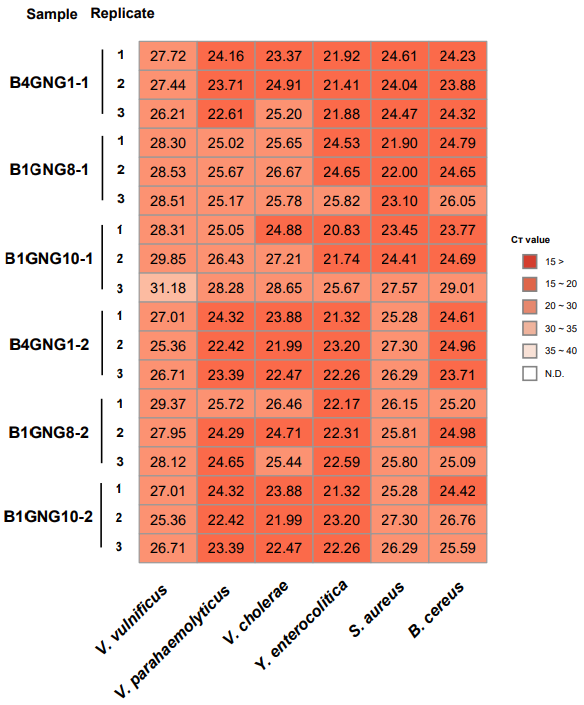


**Fig. S3. Cont.**

**E**


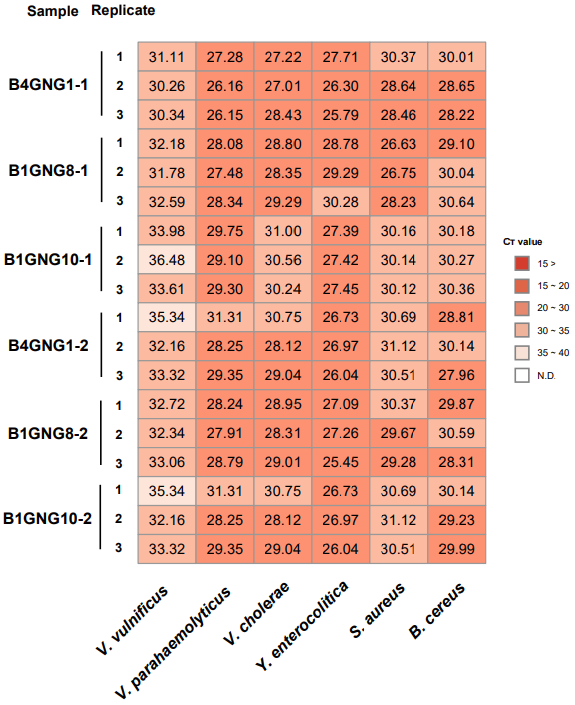

Supplement: Supplementary file 1 [file Data_Sheet_1.docx]
